# Supplementary material for: Geographic information system and information visualization capacity building: Successful polio eradication and current and future challenges in the COVID-19 era for the World Health Organization’s African region
Source: PLoS One. 2024 Aug 15;19(8):e0307001. doi: 10.1371/journal.pone.0307001 (PMC11326561; doi:10.1371/journal.pone.0307001)
Supplement: Attachment — Submitted filename: PLOS ONE GIS.docx [file pone.0307001.s001.docx]

The manuscript on **“Geographic information system and information visualization: past successes and future challenges in capacity building for the World Health Organization’s African region”** is interesting and highlights a very important tool for public health events and emergency prevention and control.

It is relevant and well laid out in terms of the critical elements that describe the utility of the WHO AFRO GIS.

However, there are a few concerns which are worth considering by the authors.

There is a disconnect between the title, the background and the objectives and some sections of the paper.

The manuscript sought to describes the components of the WHO AFRO GIS and how it was used by the established centre for polio eradication efforts in Africa. It also sought to examine successes and prospective challenges in the COVID-19 era and how the 95 GIS Centre is addressing these ongoing public health needs in Africa.

Though the WHO AFRO GIS was initially focused on polio eradication efforts, the authors alluded to the fact that it has been used for other events as well. However, the bulk of the introduction section was focused on describing polio and its eradication as though the entire manuscript was on polio. I suggest the introduction is revised to focus on GIS, its importance in disease prevention and control and how it has been used elsewhere and in Africa. This should also be done for the abstract as well.

The title of the manuscript did not mention polio. However, a chunk or almost all of the introduction section is on polio.

Also, the conclusion of the paper seems remote from the stated objectives. The recommendations are also not based on the elements in the paper but like the conclusion, appear general on one hand and also focused on polio.

Minor Comments

Line 185: Change ~ 90% to “approximately 90%”

Line 207: The subheading “2019-present” could be improved to make it more explanatory

Line 295-299: Sentence too long and unclear

Line 294-304: The whole paragraph appears to be concentrated on IPV transition which is not the main focus of this paper. I suggest this paragraph is removed.
