# Supplementary material for: Geographic information system and information visualization capacity building: Successful polio eradication and current and future challenges in the COVID-19 era for the World Health Organization’s African region
Source: PLoS One. 2024 Aug 15;19(8):e0307001. doi: 10.1371/journal.pone.0307001 (PMC11326561; doi:10.1371/journal.pone.0307001)
Supplement: Attachment — Submitted filename: Kipterer PLOS1 Response 022824.docx [file pone.0307001.s002.docx]

**Response to Reviewer/Editor Comments**

**PONE-D-23-21989**

**Geographic information system and information visualization: past successes and future challenges in capacity building for the World Health Organization’s African region**

When submitting your revision, we need you to address these additional requirements.

1. Please ensure that your manuscript meets PLOS ONE's style requirements, including those for file naming. The PLOS ONE style templates can be found at

https://journals.plos.org/plosone/s/file?id=wjVg/PLOSOne_formatting_sample_main_body.pdf and

<https://journals.plos.org/plosone/s/file?id=ba62/PLOSOne_formatting_sample_title_authors_affiliations.pdf>

***Response: Done.***

2. Thank you for stating the following financial disclosure:

"Funding for the WHO AFRO GIS is provided by the World Health Organization, Geneva, Switzerland, and Bill & Melinda Gates Foundation, Seattle, Washington, USA."

Please state what role the funders took in the study. If the funders had no role, please state: "The funders had no role in study design, data collection and analysis, decision to publish, or preparation of the manuscript." If this statement is not correct you must amend it as needed.

Please include this amended Role of Funder statement in your cover letter; we will change the online submission form on your behalf.

***Response: Please add the following text re the role of the funder: “The Bill & Melinda Gates Foundation provided human resource support during the trainings. Both funders were involved in study design, data collection and analysis, decision to publish, and preparation of the manuscript.”***

3. Thank you for stating the following in the Acknowledgments Section of your manuscript:

"Editorial support was provided by Amanda Justice and Geoff Marx of BioScience Communications, New York, New York, USA, funded by the Bill & Melinda Gates Foundation, Seattle, Washington, USA."

Funding information should not appear in the Acknowledgments section or other areas of your manuscript. We will only publish funding information present in the Funding Statement section of the online submission form.

Please remove any funding-related text from the manuscript and let us know how you would like to update your Funding Statement. Currently, your Funding Statement reads as follows:

"Funding for the WHO AFRO GIS is provided by the World Health Organization, Geneva, Switzerland, and Bill & Melinda Gates Foundation, Seattle, Washington, USA."

Please include your amended statements within your cover letter; we will change the online submission form on your behalf.

***Response: Funding Statement should read as follows: “Funding for the WHO AFRO GIS is provided by the World Health Organization, Geneva, Switzerland, and the Bill & Melinda Gates Foundation. Editorial support was provided by Amanda Justice and Geoff Marx of BioScience Communications, New York, New York, USA, funded by the Bill & Melinda Gates Foundation.”***

4. In your Data Availability statement, you have not specified where the minimal data set underlying the results described in your manuscript can be found. PLOS defines a study's minimal data set as the underlying data used to reach the conclusions drawn in the manuscript and any additional data required to replicate the reported study findings in their entirety. All PLOS journals require that the minimal data set be made fully available. For more information about our data policy, please see http://journals.plos.org/plosone/s/data-availability.

Upon re-submitting your revised manuscript, please upload your study’s minimal underlying data set as either Supporting Information files or to a stable, public repository and include the relevant URLs, DOIs, or accession numbers within your revised cover letter. For a list of acceptable repositories, please see http://journals.plos.org/plosone/s/data-availability#loc-recommended-repositories. Any potentially identifying patient information must be fully anonymized.

Important: If there are ethical or legal restrictions to sharing your data publicly, please explain these restrictions in detail. Please see our guidelines for more information on what we consider unacceptable restrictions to publicly sharing data: http://journals.plos.org/plosone/s/data-availability#loc-unacceptable-data-access-restrictions. Note that it is not acceptable for the authors to be the sole named individuals responsible for ensuring data access.

We will update your Data Availability statement to reflect the information you provide in your cover letter.

***Response: This has already been shared and data were downloaded:*** [***https://whonghub.org/faroukh/92/1537#/table***](https://whonghub.org/faroukh/92/1537#/table) ***(Username: faroukh; Password: Welcome12345). Text has been updated. [Lines 371-4]***

5. We note that Figures 1 to 3 in your submission contain map/satellite images which may be copyrighted. All PLOS content is published under the Creative Commons Attribution License (CC BY 4.0), which means that the manuscript, images, and Supporting Information files will be freely available online, and any third party is permitted to access, download, copy, distribute, and use these materials in any way, even commercially, with proper attribution. For these reasons, we cannot publish previously copyrighted maps or satellite images created using proprietary data, such as Google software (Google Maps, Street View, and Earth). For more information, see our copyright guidelines: http://journals.plos.org/plosone/s/licenses-and-copyright.

We require you to either (1) present written permission from the copyright holder to publish these figures specifically under the CC BY 4.0 license, or (2) remove the figures from your submission:

(1) You may seek permission from the original copyright holder of Figures 1 to 3 to publish the content specifically under the CC BY 4.0 license.

We recommend that you contact the original copyright holder with the Content Permission Form (http://journals.plos.org/plosone/s/file?id=7c09/content-permission-form.pdf) and the following text:

“I request permission for the open-access journal PLOS ONE to publish XXX under the Creative Commons Attribution License (CCAL) CC BY 4.0 (http://creativecommons.org/licenses/by/4.0/). Please be aware that this license allows unrestricted use and distribution, even commercially, by third parties. Please reply and provide explicit written permission to publish XXX under a CC BY license and complete the attached form.”

Please upload the completed Content Permission Form or other proof of granted permissions as an ""Other"" file with your submission.

In the figure caption of the copyrighted figure, please include the following text: “Reprinted from [ref] under a CC BY license, with permission from [name of publisher], original copyright [original copyright year].”

(2) If you are unable to obtain permission from the original copyright holder to publish these figures under the CC BY 4.0 license or if the copyright holder’s requirements are incompatible with the CC BY 4.0 license, please either i) remove the figure or ii) supply a replacement figure that complies with the CC BY 4.0 license. Please check copyright information on all replacement figures and update the figure caption with source information. If applicable, please specify in the figure caption text when a figure is similar but not identical to the original image and is therefore for illustrative purposes only.

The following resources for replacing copyrighted map figures may be helpful:

USGS National Map Viewer (public domain): http://viewer.nationalmap.gov/viewer/

The Gateway to Astronaut Photography of Earth (public domain): http://eol.jsc.nasa.gov/sseop/clickmap/

Maps at the CIA (public domain): https://www.cia.gov/library/publications/the-world-factbook/index.html and https://www.cia.gov/library/publications/cia-maps-publications/index.html

NASA Earth Observatory (public domain): http://earthobservatory.nasa.gov/

Landsat: http://landsat.visibleearth.nasa.gov/

USGS EROS (Earth Resources Observatory and Science (EROS) Center) (public domain): http://eros.usgs.gov/#

Natural Earth (public domain): <http://www.naturalearthdata.com/>

***Response: Figure 1 has been revised and does not require permission. Sources for Figures 1 and 4 have been added to legends. New Figures 2 and 5 (formerly 3) are derived from GeoPoDe, an open-source, public data repository for geospatial reference datasets, and does not require permission. However, we have requested and received permission to be safe. (Note: GeoPoDe is a public web data repository for geospatial datasets available by country. The platform allows public health officials and partner organizations to visualise geospatial data on an interactive map and download the data in multiple formats. All datasets contained in GeoPode are available to non-profit or humanitarian applications and are “operational” in nature. The datasets do not constitute authoritative, government-sanctioned reference data.)***

Additional Editor Comments:

•	Please review and consider all the comments and suggestions by both reviewers to provide more clarity and context within the manuscript.

***Response: Done***

•	Both reviewers have also highlighted the focus on Polio in the current draft and the need to revise the title and text to reflect the aim of this review as stated in Lines 92-95? Is the manuscript about GIS and Polio alone or other diseases as well? You may revise the title of the manuscript accordingly showing "past" Polio Successes." and challenges "post covid" for GIS utilization for other disease outbreaks. Any examples of how the GIS system has supported any other diseases? (how is the AFRO GIS Centre addressing these ongoing public health needs?)

***Response: The title has been revised: “Geographic information system and information visualization capacity building: successful polio eradication, and current and future challenges in the COVID-19 era.”***

***Examples of GIS applications in routine immunization to improve vaccination coverage in the Lake Chad region,and many WHO/AFRO countries are enumerated in an earlier publication: Akpan GU et al. Conclusions of the African Regional GIS Summit (2019): using geographic information systems for public health decision-making. BMC Proc 16 (Suppl 1), 3 (2022). https://doi.org/10.1186/s12919-022-00233-y.***

•	The current title also includes "capacity building". The conclusion is specific for Polio and GPEI, is this capacity building for WHO GIS Polio teams or for country field staff for Polio and other diseases as well?

***Response: Capacity building is for polio and other diseases, as well. As noted, title has been revised.***

•	In line with the recommendation on the title, please update the abstract and the conclusion to ensure alignments. Note also that the conclusion only mentions Polio and nothing about other diseases outbreaks.

***Response: We have revised the last sentence in the abstract (“It also examines current and prospective challenges regarding other disease outbreaks in the COVID-19 era and how the AFRO GIS Centre is addressing these ongoing public health needs.”) to address this issue [Lines 42-3]. The end of the Conclusion has been revised: “…with the goal of faster detection and response to polio and other disease outbreaks in the COVID-19 era.” [Line 363]***

•	Lines 169-171 documents polio vaccination by WHO AFRO GIS teams? Does the AFRO GIS deploy vaccination teams or is this by the teams recruited by the Governments? Is there a reference for this?

***Response: It is the role of WHO/AFRO to respond through vaccination, and deployment is jointly made by WHO and the respective government. Additional references have been added.*** <https://www.afro.who.int/health-topics/polio/polio-update-on-country-situations/nigeria-and-the-lake-chad-countries>; <https://polioeradication.org/countries/lake-chad-region/>

•	The section on use of GIS beyond Polio (Lines 245-276) is a clear example of Polio transition in action. Please include a line or two on Polio transition.

***Response: We have added the following text: “The integration of polio transition processes involves the bringing together of various stakeholders and resources to ensure a smooth and effective transition. This includes integrating polio surveillance systems into existing disease surveillance networks, incorporating polio vaccinations into routine immunization programs, and merging the expertise and infrastructure developed for polio eradication with broader public health initiatives.” [Lines 291-5]***

•	Please also revise to correct sentences and punctuations as may be required for consistency E.g.: Line 215 & 21: Train (Orient) participants "on" while Line 219: Train participants "in".

[Note: HTML markup is below. Please do not edit.]

***Response: Done***

Reviewers' comments:

Reviewer's Responses to Questions

Comments to the Author

1. Is the manuscript technically sound, and do the data support the conclusions?

The manuscript must describe a technically sound piece of scientific research with data that supports the conclusions. Experiments must have been conducted rigorously, with appropriate controls, replication, and sample sizes. The conclusions must be drawn appropriately based on the data presented.

Reviewer #1: Partly

Reviewer #2: Partly

***Response: Revisions made as specified in comments.***

2. Has the statistical analysis been performed appropriately and rigorously?

Reviewer #1: N/A

Reviewer #2: N/A

***Response: N/A***

3. Have the authors made all data underlying the findings in their manuscript fully available?

The PLOS Data policy requires authors to make all data underlying the findings described in their manuscript fully available without restriction, with rare exception (please refer to the Data Availability Statement in the manuscript PDF file). The data should be provided as part of the manuscript or its supporting information or deposited to a public repository. For example, in addition to summary statistics, the data points behind means, medians and variance measures should be available. If there are restrictions on publicly sharing data—e.g. participant privacy or use of data from a third party—those must be specified.

Reviewer #1: Yes

Reviewer #2: Yes

***Response: Thanks***

4. Is the manuscript presented in an intelligible fashion and written in standard English?

PLOS ONE does not copyedit accepted manuscripts, so the language in submitted articles must be clear, correct, and unambiguous. Any typographical or grammatical errors should be corrected at revision, so please note any specific errors here.

Reviewer #1: Yes

Reviewer #2: Yes

***Response: Thanks***

Reviewer #1: In summary this manuscript outlines the development and expansion of the use of GIS systems in the African WHO region for primarily polio eradication. This is a very important technological development both for polio eradication and future disease surveillance and intervention monitoring. The manuscript provides narrative of the progress to date, highlighting particular GIS gaps (i.e. it is not a scientific research article). However, to a reader not involved in this GIS network, more details are required to understand the processes. The authors also present data from a 2019 survey on national GID capacity but more details are required as to who was surveyed and whether follow-up data will be collected to measure progress. It would be much clearer if different visualizations were presented for a particular outbreak that the network has worked on e.g., The authors conclude the GIS system was important for achieving certification of WPV1 in the region, it would be helpful to see some examples of the different GIS layers that contributed to this.

***Response: There are no layers involved; it was achieved through surveillance visits, with evidence of visits at the health facility level with GPS enabled on submission of visit checklists. The use of GIS enables technologies in vaccination tracking systems and the use of the AVADAR electronic surveillance checklist serves as evidence (see:*** [***https://doi.org/10.29245/2578-3009/2021/S2.1101***](https://doi.org/10.29245/2578-3009/2021/S2.1101)***,*** ***http://dx.doi.org/10.16966/2471-8211.151***, **doi: 10.11604/pamj.supp.2022.42.1.33788, *10.2196/18950), https://doi.org/10.1186/s12889-018-6187-x).***

Minor comments:

Abstract:

‘Currently, 95% of reported polio cases are in under-vaccinated populations’ it is not clear what you mean by this given many children will not have received type 2 OPV since 2016.

***Response: This statement has been deleted since we can’t find the definitive reference. [Lines 51-3]***

Introduction

Line 49, I think you mean WPV1 free?

***WPV 2 is correct.***

Line 66, By vaccine-associated do you mean vaccine-derived?

***Response: You are correct, and this has been revised.***

Fig 1 why do you choose this specific time period?

***Response: This was based on the timing of manuscript development. The figure has been revised to cover 29 October 2022 to 30 October 2022.***

The introduction would benefit with a few sentences describing withdrawal of type 2 from OPV in 2016 and the following declining immunity across the region, facilitating cVDPV2 spread. A little more detail required on the rollout of nOPV2 would help the reader.

***Response: New text has been added: “The rollout of nOPV2 in the African region is aimed at addressing the outbreak of polio, strengthening immunization efforts, reducing the transmission of cVDPV2, and contributing to the efforts of the GPEI [13, 14]. This initiative represents a significant milestone in combating polio outbreaks, particularly in the countries of the Lake Chad Basin region, which have been significantly impacted by the disease [15]. The introduction of nOPV2 in Nigeria and Liberia is a crucial step towards effectively responding to the ongoing cVDPV2 outbreaks and preventing further transmission of the virus [16].” [Lines 86-92]***

Figure 2 – Requires more details in the legend – how is the data obtained? Which part of Nigeria is shown?

***Response: Fig 2 has been replaced by a new and the legend has been revised with more detail: “Fig 2. Screen capture from NigerianGhana (northern region) GeoPoDe display showing location of health facilities and towns. The data are obtained by WHO from the ministry of health and bureaus of statistics, and are incorporated in the GeoPoDe by the WHO in support of ministry of health decision-making.” [Lines 137-40]***

Paragraph 114 – ‘Components of the WHO AFRO geographic information system’. This section is hard to follow. Perhaps include a diagram to show if any of these platforms interact? More description required to understand what ISS, eSurv and AVADAR are – how do they interact with other GIS platforms? Perhaps show an example from each? It would be helpful to highlight in the diagram what new spatial data is the GIS Centre helping to collect compared to visualising standard surveillance data. It is not clear what PowerBI is used for and what ARC GIS is used for.

***Response: New Figs 3 and 4, and text have been added: “Integrated Supportive Supervision and eSURV are mobile health solutions for effective supervision and surveillance in healthcare. Integrated Supportive Supervision utilizes electronic checklists on Android phones for active case finding and routine immunization supervision by the WHO and government staff [25]. Electronic surveillance ensures government surveillance agents conduct active searches using mobile phones in health facilities and communities [26]. Auto-Visual AFP Detection and Reporting is a community-based surveillance solution that employs the use of a mobile app combining visual, audio, and Short Message Service reporting to enhance AFP surveillance [27]. It aims to improve AFP case detection and reporting, enhance reporting timeliness, ensure “Zero” reporting of AFP cases, provide real-time case information, and increase community participation and ownership in surveillance efforts.***

***The data collected and stored in the WHO servers through these solutions are connected to ArcGIS Online and Power BI for analyses that involve mapping and visualizations, with program indicators of surveillance visualized in a dashboard. Visualization analytics are used by the program staff to identify gaps in surveillance, where areas not visited by active case search and routine surveillance personnel are quickly identified for action, while the AFP cases reported by AVADAR are also integrated in the AFP database for follow-up.” [Lines 148-72]***

Figure 3– what data is going into this dashboard? How are nomadic populations ‘tracked’ it could be interpreted as unethical tracking of certain people so please explain.

***Response: The dashboard represents the key attribute of the nomadic population during the data collection survey, where their locations, based on the season, were captured to aid vaccination teams (health workers) reach the children for polio campaigns, as the population presence in such locations is seasonal. It is nomad mapping, but not tracking. Fig 5 (formerly Fig 3) has been replaced with a new figure.***

Figure 4 – what is the y axis? % missing or having. Please provide more details on the survey – questions in the appendix? Who participated?

***Response: The y axis in this figure represents the percentage of responses in agreement with the question captured in the x axis. More detail has been added to the legend: “A: Survey responses from WHO AFRO GIS country teams regarding documentation of healthcare facility locations; provision of office resources for GIS staff, equipment, and software; and availability of trained GIS staff. Survey questions focused on geographic details, personal information, foundations of GIS, baseline data and standards, capacity building, services and applications, evaluation, challenges of GIS implementation, and lessons learned.” [Lines 228-32]***

Train the trainer – who are these trainers, how are they located across countries? Who are those being trained?

***Response: Text has been added: “The WHO/AFRO GIS Centre staff train data managers from ministries of health and WHO country offices, who, in turn, cascade the training to the subnational level, ie, district-level staff.” [Lines 251-3]***

Line 232 – population and demographic data – based on what data source? Most recent census or other?

***Response: 2020 WorldPop datasets. Text and ref have been added: “…based on 2020 WorldPop datasets [23, 35].” [Lines 276-7]***

Line 262 Consolidated health facility master list – this is a very exciting development. Is it possible to report on how complete this database is? What are the barriers to obtaining more data etc? What proportion of countries have supplied data….

***Response: Development of this master list is an ongoing process. Another paper by colleagues of ours focusing on this topic is expected to be published shortly (Nshuti MAB et al, JMIR Public Health Surveill).***

IPV – has the GIS Centre been involved in evaluating the (few) IPV campaigns?

***Response: The GIS Centre has not been involved in these campaigns and the relevant paragraph has been deleted.***

Reviewer #2: Authors should align the title, the main text and the conclusion and recommendations of this paper. The paper in its current form seems to be too much focused on polio even though its intention is to highlight the application ion WHO AFRO GIS tool in prevention and control of not only polio but other diseases.

***Response: As noted previously, the title, main text, and conclusion have been revised to reflect these concerns.***

Reviewer #3: The manuscript on “Geographic information system and information visualization: past successes and future challenges in capacity building for the World Health Organization’s African region” is interesting and highlights a very important tool for public health events and emergency prevention and control.

It is relevant and well laid out in terms of the critical elements that describe the utility of the WHO AFRO GIS.

However, there are a few concerns which are worth considering by the authors.

There is a disconnect between the title, the background and the objectives and some sections of the paper.

The manuscript sought to describes the components of the WHO AFRO GIS and how it was used by the established centre for polio eradication efforts in Africa. It also sought to examine successes and prospective challenges in the COVID-19 era and how the 95 GIS Centre is addressing these ongoing public health needs in Africa.

Though the WHO AFRO GIS was initially focused on polio eradication efforts, the authors alluded to the fact that it has been used for other events as well. However, the bulk of the introduction section was focused on describing polio and its eradication as though the entire manuscript was on polio. I suggest the introduction is revised to focus on GIS, its importance in disease prevention and control and how it has been used elsewhere and in Africa. This should also be done for the abstract as well.

***Response: We have endeavored to make edits throughout the manuscript to address these comments.***

The title of the manuscript did not mention polio. However, a chunk or almost all of the introduction section is on polio.

***Response: As noted previously, the title has been revised.***

Also, the conclusion of the paper seems remote from the stated objectives. The recommendations are also not based on the elements in the paper but like the conclusion, appear general on one hand and also focused on polio.

***Response: As noted previously, the conclusion has been revised.***

Minor Comments

Line 185: Change ~ 90% to “approximately 90%”

***Response: Done***

Line 207: The subheading “2019-present” could be improved to make it more explanatory

***Response: Note: we have left this as is to match the previous subhead for consistency (ie, “2017–2019”), but havereplacethe hyphen with “to.”***

Line 295-299: Sentence too long and unclear

***Response: Note: based on other comments, this entire paragraph has been deleted. [Lines 344-54]***

Line 294-304: The whole paragraph appears to be concentrated on IPV transition which is not the main focus of this paper. I suggest this paragraph is removed.

***Response: Again, this paragraph has been deleted. [Lines 344-54]***
